# A segregation analysis of testicular cancer based on Norwegian and Swedish families.

**DOI:** 10.1038/bjc.1997.185

**Published:** 1997

**Authors:** K. Heimdal, H. Olsson, S. Tretli, S. D. FossÃ¥, A. L. BÃ¸rresen, D. T. Bishop

**Affiliations:** Department of Genetics, Institute for Cancer Research, The Norwegian Radium Hospital, Oslo.

## Abstract

Clustering of testicular cancer cases in families is well known, although the aetiology is not. We present the results of a segregation analysis performed with the algorithm Pointer on familial data on 978 Scandinavian patients with testicular cancer. The segregation analysis favoured the involvement of major gene effects over models incorporating solely polygenic effects in testicular cancer aetiology. Overall, a recessive model best fits the family observations with an estimated gene frequency of 3.8% and a lifetime risk for homozygous men of developing the disease of 43%. This implies that 7.6% of men in the general population will be carriers of the mutant allele and that 0.1% would be homozygote and are, therefore, at high risk of developing the cancer. The testicular cancer incidence has changed greatly during the last generation. Also, the lethality of the disease has changed because of the introduction of new therapy. As failure to take account of such time trends might lead to inappropriate evidence for a recessive model, the analyses were repeated under different assumptions. The analyses favoured a recessive model of inheritance under all assumptions tested. However, the assumptions underlying the analyses are complex and, as this is the first segregation analysis of testicular cancer, the results must be interpreted cautiously.


British Journal of Cancer (1997) 75(7), 1084–1087
© 1997 Cancer Research Campaign

### A segregation analysis of testicular cancer based on Norwegian and Swedish families

K Heimdal[1,2], H Olsson[3], S Tretli[4], SD Fosså[2], A-L Børresen[1] and DT Bishop[5]

[1]Department of Genetics, Institute for Cancer Research and [2]Department of Oncology, The Norwegian Radium Hospital, Oslo, Norway; [3]Department of Oncology, University Hospital Lund, Sweden; [4]The Norwegian Cancer Registry, Oslo, Norway; [5]Imperial Cancer Research Fund, Leeds, UK

**Summary** Clustering of testicular cancer cases in families is well known, although the aetiology is not. We present the results of a segregation analysis performed with the algorithm Pointer on familial data on 978 Scandinavian patients with testicular cancer. The segregation analysis favoured the involvement of major gene effects over models incorporating solely polygenic effects in testicular cancer aetiology. Overall, a recessive model best fits the family observations with an estimated gene frequency of 3.8% and a lifetime risk for homozygous men of developing the disease of 43%. This implies that 7.6% of men in the general population will be carriers of the mutant allele and that 0.1% would be homozygote and are, therefore, at high risk of developing the cancer. The testicular cancer incidence has changed greatly during the last generation. Also, the lethality of the disease has changed because of the introduction of new therapy. As failure to take account of such time trends might lead to inappropriate evidence for a recessive model, the analyses were repeated under different assumptions. The analyses favoured a recessive model of inheritance under all assumptions tested. However, the assumptions underlying the analyses are complex and, as this is the first segregation analysis of testicular cancer, the results must be interpreted cautiously.

**Keywords:** testicular cancer; genetics; segregation analysis

Segregation analysis is a statistical technique which attempts to explain the causes of family aggregation of disease (Morton et al, 1983). Basically, a major gene, polygenes and unmeasured environmental exposures are assumed to contribute to susceptibility to disease and, on the basis of the observed pattern of disease within families, different explanations of the family aggregation are compared. The technique is most informative when the family material consists of the relatives of a systematically collected series of cases from a population-based register. Such studies have resulted in the identification of putative major gene effects for susceptibility to cancer at several sites, including the breast (e.g. Claus et al, 1991), ovary (Houlston et al, 1991) and bowel (Houlston et al, 1995). These results have been verified at least in part by the successful linkage mapping of genes that contribute to the susceptibility (Hall et al, 1990; Peltomaki et al, 1993).

Testicular germ cell cancer is widely believed to be caused mainly by environmental factors operating in utero or in early childhood (Oliver, 1990). However, familial clustering is well known, and there is an increased relative risk for male first-degree relatives of testicular cancer cases. The relative risk to brothers has been estimated to be as high as 6–10 (Tollerud et al, 1985; Forman et al, 1992; Goldgar et al, 1994; Heimdal et al, 1996). This is higher than for most common cancers studied and highlights the probable importance of genetic factors in disease causation (Cannon-Albright et al, 1991). In contrast to published multicase families for the common cancers, families usually have only two members affected by testicular cancer, and simple inspection of the pedigrees is not sufficient to establish the mode of inheritance.

Two factors complicate the interpretation of the incidence of familial testicular tumours. Firstly, the incidence rates of testicular cancer have changed greatly and, secondly, treatment has changed the lethality of the disease during the last generation (Oliver, 1990). In the parental generation (before 1960 approximately), there was essentially no effective treatment for metastatic testicular cancer, and the fertility of the group of affected individuals would be greatly reduced. Thus, men with testicular cancer diagnosed before 1960 would be less likely to produce offspring or, equivalently, the sons born in the 1960s would be less likely to have an affected parent than would be predicted on the basis of population rates. Subsequently, probands (ascertained during the 1980s) were given effective multimodal treatment. This group therefore has only marginally reduced fertility. These two factors present appreciable complications to a segregation analysis of testicular cancer.

We present the results of the first segregation analysis of testicular cancer. This analysis, using the software Pointer, is based on the families of 978 Scandinavian patients treated at the Norwegian Radium Hospital and in Lund, Sweden, over a 10-year period.

### MATERIALS AND METHODS

### Ascertainment of families

The family material on which the segregation analysis was based consisted of the relatives of all available patients treated at the Norwegian Radium Hospital and in Lund for testicular germ cell tumour from 1981–91 as described elsewhere (Heimdal et al, 1996). The two institutions are responsible for the post-orchiectomy

*Received 4 December 1995*
*Revised 14 October 1996*
*Accepted 16 October 1996*

*Correspondence to:* K Heimdal, Department of Medical Genetics, Haukeland University Hospital, N-5021 Bergen, Norway

**Table 1** The definition of the five liability classes assumed in the segregation analysis and the corresponding estimated cumulative incidence of testicular cancer to midpoint of the age interval (columns 1–2). The cumulative incidence has been calculated from population incidences of testicular cancer in Norway (see text). The estimated penetrances from segregation analysis are also given in this table (columns 3–4). These penetrances also relate to the midpoint of the specified agent force

| Definition of liability class | Cumulative incidence to midpoint | Recessive susceptible | Heterozygote/homozygote normal |
|---|---|---|---|
| 1. Proband generation: men aged 15–34 | 0.00150 | 0.27 | 0.0011 |
| 2. Proband generation: men aged 35–54 | 0.00390 | 0.39 | 0.0034 |
| 3. Proband generation: men aged 55 and above | 0.00530 | 0.43 | 0.0047 |
| 4. Parental generation: men aged 35–54 | 0.00120 | 0.24 | 0.0009 |
| 5. Parental generation: men aged 55 and above | 0.00190 | 0.29 | 0.0015 |

treatment of all testicular cancer in their catchment areas. Family history was collected through a questionnaire-based survey of the patients. All patients treated at the two institutions during this period who were alive and could be located were invited to complete the questionnaire asking for information on testicular cancer in their first-degree relatives, the ages of onset for cases and current ages for unaffected relatives.

Eighty-four per cent (978 out of 1159 patients) of the patients returned questionnaires with family information. The majority of patients about whom no family information was available had died before this study was conducted. Testicular cancer in a first-degree relative was reported by 30 patients (Heimdal et al, 1996). All diagnoses of testicular germ cell tumour in probands and relatives have been verified in the Norwegian or Swedish Cancer Registries and/or by histological reports. From the 978 probands, five fathers and two father–son pairs (doubly ascertained) and 17 brother–brother pairs, six of which were doubly ascertained, were found to be affected with testicular cancer. In the analyses conducted here, no family had more than two affected individuals.

### Statistical analysis

Segregation analysis was performed using the software Pointer (Lalouel and Morton, 1981). Pointer assumes the mixed model of inheritance, that is, segregation is explained by a major gene, a polygenic background and unmeasured environmental exposures. The effects of these factors are assumed to be additive on the liability scale. Pointer does not allow for the joint analysis of the ascertained families but rather considers an extended pedigree as being made up of its nuclear family components. These nuclear families are related to the extended pedigree by conditioning on their relatedness to each other.

The parameterization of the mode of inheritance takes advantage of the major assumption of the model, which is that all factors act on the liability scale. For the major gene component, the model assumes two alleles at a single locus ($A$, $a$) with $A$ being associated with increased risk of testicular cancer. Individuals with an '*aa*' genotype are assumed (without loss of generality) to have a value of 0 on the liability scale, while the disease-associated homozygote has a value of $t$ (> 0) and the heterozygote $d^*t$. For the heterozygote to have an effect on penetrance that is intermediate between the two homozygotes, $d$ is constrained by $0 < d < 1$. The allele frequency of $A$ is $q$, and Hardy–Weinberg equilibrium is assumed. Finally, the residual polygenic heritability is modelled by $H$, the proportion of variance around these values for the major genes which is determined by polygenic inheritance under the assumption that deviations from the stated values for the major gene component are normally distributed. These assumptions are sufficient to define the threshold value $T$ for the liability so that any male whose liability exceeds $T$ is affected with testicular cancer. $T$ is estimated for a given set of parameter values from the cumulative incidence rate of testicular cancer.

As both hospitals in this study treat all cases diagnosed in their geographical regions, families with the proband as a child were coded for Pointer as having ascertainment with a high value of $\pi$, the ascertainment probability. There were 970 such families, seven of them with an affected parent. The families with the proband as parent were coded as complete ascertainment, that is their ascertainment followed directly after the identification of the proband. There were 454 such families with 2697 children.

In an attempt to accommodate the complicating factors of changing fertility (through treatment improvements) and incidence, men were classified to one of five liability classes depending on their generation within the pedigree (proband generation vs parent of proband generation) and their current age or age at testicular cancer onset [15–34 years of age (proband generation only), 35–54 years of age and greater than 54 years of age] (see Table 1). Allowing for different liability classes by generation should accommodate the changing rates of testicular cancer on the population level. Cumulative incidence rates for these five liability classes were calculated on the basis of published 10-year incidence rates for testicular cancer in Norway during 1982–91 (Table 1). The lack of registry data for the previous generations further complicates the assessment of cumulative incidence rates for the liability classes of the parental generation. To circumvent this problem, the analyses were repeated assuming that incidence rates in the parental generation were one-third of, one-half of and equal to the 1982–91 incidence rates. The extremes of these assumptions are thought to cover the range of incidence rates since cancer registration began in the 1950s. At that time, there were approximately one-third the reported number of cases of testicular cancer annually compared with current times. These assumptions allow the estimation of a separate threshold $T$ for each liability class. Women and men under the age of 15 years were coded to indicate their lack of predisposition to testicular cancer.

To allow for the effect of parental disease on fertility, fathers of probands were assumed to be not at risk of testicular cancer before the age of 35 years. There were no such fathers in the sample and the youngest diagnosis in a father was 36 years of age. Fathers were assumed to have incidence rates of testicular cancer identical to the general population after age 35 years as the majority of their offspring would have been born before that age. Analyses were conducted conditional on parental disease status in a further effort to compensate for fertility differences in affected men.

© Cancer Research Campaign 1997

Table 2 The comparison of likelihoods under the mixed model with the estimated parameters under that model. Parameter values in parentheses are fixed at that value under that assumed model. The log-likelihood difference is based on comparisons with the mixed model for which all components are included in the model

| Model | d | t | q | H | 2 * Ln difference |
|---|---|---|---|---|---|
| Complete | 0.0 | 2.42 | 0.038 | 0.00 | – |
| Recessive major gene | (0.0) | 2.42 | 0.038 | (0.0) | 0.00 |
| Dominant major gene | (1.0) | 1.60 | 0.003 | (0.0) | 5.18 |
| Additive major gene | (0.5) | 2.08 | 0.07 | (0.0) | 4.54 |
| Polygenic | – | – | – | 0.43 | 7.16 |
| Sporadic | – | – | – | – | 38.51 |

Analyses were also repeated for a range of values of π with the ascertainment probability from 0.4 to 0.9. Varying π did not have substantial effects on the results (data not shown). Analyses reported here are computed with a value of π of 0.52, which is the estimated value of π when there are 17 brother–brother pairs, six of which are doubly ascertained (Morton et al, 1983).

### RESULTS

Table 2 shows the results of the segregation analysis assuming that the parents of probands had incidence rates one-half of that of the proband generation. Segregation analysis suggests that a recessive model best fits the collected families (Table 2). For comparison of models, we report twice the natural logarithm difference between the model under consideration and the complete model, that is the model with the major gene and polygenic components being jointly estimated from the total dataset and other assumed models. For the complete model, the estimate of the polygenic heritability was zero while the major gene model favoured a recessive gene.

Among the major gene models, a recessive model had a value of twice the natural logarithm greater than 4.5 more than the value for either the dominant or an additive model in which heterozygotes had intermediate penetrance between that of each of the two normal homozygotes. A purely polygenic model had a value of twice the natural logarithm of 7.16 less than the recessive model. For the comparisons given in Table 2, the recessive model therefore was considered parsimonious.

Under this recessive model the estimated gene frequency was 3.8% (Table 2). This implies that 7.6% of the men would be carriers of such a mutant allele but only those who were homozygotes would be at high risk of testicular cancer. Under this model, homozygote men would have a 43% lifetime risk of developing testicular cancer. Furthermore, about 25% of testicular cancer cases diagnosed before the age of 35 years in the proband generation would be attributed to this susceptibility, 14% of cases between 35 and 54 years and 12% after the age of 55 years. For the parental generation, the susceptibility would explain slightly higher proportions of all cases (because the population incidence rates were lower), i.e. 29% of cases between 35 and 54 years and 22% of cases above the age of 55 years.

One possible explanation of a recessive mode of inheritance is that the difference in incidence rates is not well modelled by our assumption that the parental generation has incidence rates of one-half of the proband generation. We therefore repeated the analysis assuming that the incidence rates had not changed over time. Although this latter assumption does not seem feasible in the light of the number of cases reported to the cancer registries, it does allow an extreme analysis of the role of inherited susceptibility. In fact, under this assumption, a recessive model is still the most plausible explanation with an estimated frequency of the susceptibility mutation of 0.033 and a lifetime risk of testicular cancer of 49%. Model comparisons are similar to those presented here except that the distinction between the recessive models and the others is increased (to, for instance, 5.80 over the dominant model) in keeping with the failure to take into account the trend in incidence rates. At the other extreme, when the rates in the parental generation are one-third of those in the proband generation, the estimated frequency of the susceptibility allele is 0.035 with an associated lifetime risk of 46%. In summary, therefore, as varying the assumptions about the relative incidence of testicular cancer in parental and proband generations within reasonable limits did not appreciably change the best fitting models or the model comparisons, these particular estimates seem quite robust.

Analyses conducted with different values of π produced only marginally different recessive models and had similar results to those given in Table 2 for model comparisons (data not shown).

### DISCUSSION

As this is the first segregation analysis of testicular cancer, the results should be verified in other data sets. Our analysis is based on more than one-half of the total number of cases seen in Norway and all cases seen in Southern Sweden during the period in which modern treatment has been curing the great majority of testicular cancer patients. Analyses are required on data sets from other populations to compare with the findings here, especially as the scarcity of multiple-case families reduces the ability to distinguish between modes of inheritance in segregation analysis.

Segregation analysis favours the involvement of major gene effects in testicular cancer causation. Our analyses are in favour of a recessive mechanism; this conclusion seems justified in light of the lack of variation in the estimates of the model parameters when the frequency of testicular cancer was varied in the parental generation.

The results are in support of major gene models over models incorporating polygenic effects only. The analysis favours recessive gene models, and there are indications that testicular cancer may be the first common cancer caused by a relatively common recessive gene, possibly in combination with polygenic–environmental effects operating predominantly in recent generations.

Nicholson and Harland (1995), using a different approach and analysis from the current work, also favour a recessive gene that is responsible for one-third of testicular cancer cases. This is somewhat higher than our estimates. However, their estimate of the gene frequency (5%) is similar to ours using the recessive model (3.6%). Furthermore, they estimate the penetrance to be 45%. Their analysis is based on the frequency of bilateral to unilateral tumours and takes into account information that is not used here. Their conclusions should therefore be regarded as independent support for a recessive mechanism.

There are at least four studies showing the relative risk to brothers to be between 6 and 10 (Tollerud et al, 1985; Forman et al, 1992; Goldgar et al, 1994; Heimdal et al, 1996). Because of methodological difficulties, especially concerning the lethality of the disease and the resulting lack of fitness of affected individuals in former generations combined with the lack of reliable cancer registries before 1950, the estimates of risk to fathers have wider confidence limits. Published studies do, however, indicate that the

© Cancer Research Campaign 1997

increase in relative risk is greater in brothers than in fathers of testicular cancer cases (Heimdal et al, 1996). This finding is also consistent with a recessive model of inheritance.

The ability to identify genes that predispose to testicular cancer is determined by the true mode of inheritance, the number of genes involved and their relative frequencies and penetrance. Segregation analysis cannot distinguish between one and more than one major gene. However, if a single gene were involved then the magnitude of the increased risk to first-degree relatives indicates that such a gene should be readily mapped (Risch, 1990). In the first major effort to map such a gene, brothers with testicular cancer have been identified and sampled and then a genomic search was performed (Leahy et al, 1995). Evidence for a number of genetic regions has been shown but no one region is unequivocally the site of a testis cancer gene. It is of note, however, that for the regions with the highest LOD scores, evidence for linkage was strongest under recessive models. This must be taken into account in further genetic studies of this disease. Our results also illustrate that most testicular cancer is caused by other factors than major genes (polygenes and environmental factors) and emphasizes the importance of environmental factors in disease causation. Thus, the present data are in keeping with the marked increase in testicular cancer incidence rates over the past generation, which cannot be due to genetic factors alone.

Segregation analyses in other familial cancer syndromes (familial breast, breast–ovarian, HNPCC and Li Fraumeni syndromes) have indicated the involvement of rare dominant alleles (Claus et al, 1991; Houlston et al, 1995). Also, in ataxia telangiectasia, even though the syndrome is caused by the homozygous (recessive) genotype, cancer risk is increased both in homozygotes and heterozygotes (Swift et al, 1991). The current analysis cannot soundly reject the hypothesis that testicular cancer fits the general concept of genetic cancer caused by a very rare, dominant allele (Table 2). The difference, when comparing testicular cancer with other genetic cancers using the dominant model, is that the testis cancer causing allele has very low penetrance. Thus testicular cancer is usually not familial. However, we cannot reject the hypothesis that testicular cancer may be the first relatively common cancer discovered to be caused by a fairly common recessive allele.

In summary, segregation analysis supports the contention that testicular cancer is due to a recessive gene. One major concern about this analysis is the issue of time trends in testicular cancer rates, i.e. changing lethality of the disease and changing fertility of males with testicular cancer. Inappropriate accounting for such trends might lead to inappropriate evidence for a recessive model. We have investigated this and have found that modifying assumptions about testicular cancer rates does make a minor difference to the evidence for the recessive model of inheritance. However, for each different set of assumptions, a recessive model seems to offer the most reasonable explanation for the clustering of testicular cancer cases in families.

### ACKNOWLEDGEMENTS

This study was supported by the Norwegian Cancer Society. KH is a fellow of the Norwegian Cancer Society.

### REFERENCES

Cannon-Albright LA, Bishop DT, Goldgar D and Skolnick MH (1991) Genetic predisposition to cancer. *Important Adv Oncol* 39–55

Claus EB, Risch NJ and Thompson WD (1991) Genetic analysis of breast cancer in the cancer and steroid hormone study. *Am J Hum Genet* **48**: 232–242

Forman D, Oliver RTD, Brett AR, Marsh SGE, Moses JH, Bodmer JG, Chilvers CED and Pike MC (1992) Familial testicular cancer: a report of the UK family register, estimation of risk and HLA Class 1 sib-pair analysis. *Br J Cancer* **65**: 255–262

Goldgar DE, Easton DF, Cannon-Albright LA and Skolnick MH (1994) Systematic population-based assessment of cancer risk in first-degree relatives of cancer probands. *J Natl Cancer Inst* **86**: 1600–1608

Hall JM, Lee MK, Newman B, Morrow JE, Anderson LA, Huey B and King MC (1990) Linkage of early-onset familial breast cancer to chromosome 17q21. *Science* **250**: 1684–1689

Heimdal K, Olsson H, Tretli S, Flodgren P, Børresen A-L and Fosså SD (1996) Familial testicular cancer in Norway and southern Sweden. *Br J Cancer* **73**: 964–969

Houlston RS, Collins A, Slack J, Campbell S, Collins WP, Whitehead MI and Morton NE (1991) Genetic epidemiology of ovarian cancer: segregation analysis. *Ann Hum Genet* **55**: 291–299

Houlston RS, Collins A, Kee F, Collins BJ, Shields DC and Morton NE (1995) Segregation analysis of colorectal cancer in Northern Ireland. *Hum Hered* **45**: 41–48

Lalouel JM and Morton NE (1981) Complex segregation analysis with pointers. *Hum Hered* **31**: 312–321

Leahy MG, Tonks S, Moses JH, Brett AR, Huddart R, Forman D, Oliver RTD, Bishop DT and Bodmer JG (1995) Candidate regions for a testicular cancer susceptibility gene. *Hum Molec Genet* **4**: 1551–1556

Morton NE, Rao DC and Lalouel JM (1983) *Methods in Genetic Epidemiology*. S Karger: Basle

Nicholson PW and Harland SJ (1995) Inheritance and testicular cancer. *Br J Cancer* **71**: 421–426

Oliver RTD (1990) Atrophy, hormones, genes and viruses in aetiology of germ cell tumours. *Cancer Surv* **9**: 263–286

Peltomaki P, Aaltonen LA, Sistonen P, Pylkkanen L, Mecklin JP, Jarvinen H, Green JS, Jass JR, Weber JL, Leach FS, Petersen GM, Hamilton SR, De La Chapelle A and Vogelstein B (1993) Genetic mapping of a locus predisposing to human colorectal cancer. *Science* **260**: 810–812

Risch N (1990) Linkage strategies for genetically complex traits. I. Multilocus models. *Am J Hum Genet* **46**: 222–228

Tollerud DJ, Blattner WA, Fraser MC, Brown LM, Pottern L, Shapiro E, Kirkemo A, Shawker TH, Javadpour N, O'Connell K, Stutzman RE, and Fraumeni JF (1985) Familial testicular cancer and urogenital development anomalies. *Cancer* **55**: 1849–1854

Swift M, Morrell D, Massey RB and Chase CL (1991) Incidence of cancer in 161 families affected by ataxia-telangiectasia. *N Engl J Med* **325**: 1831–1836

© Cancer Research Campaign 1997 


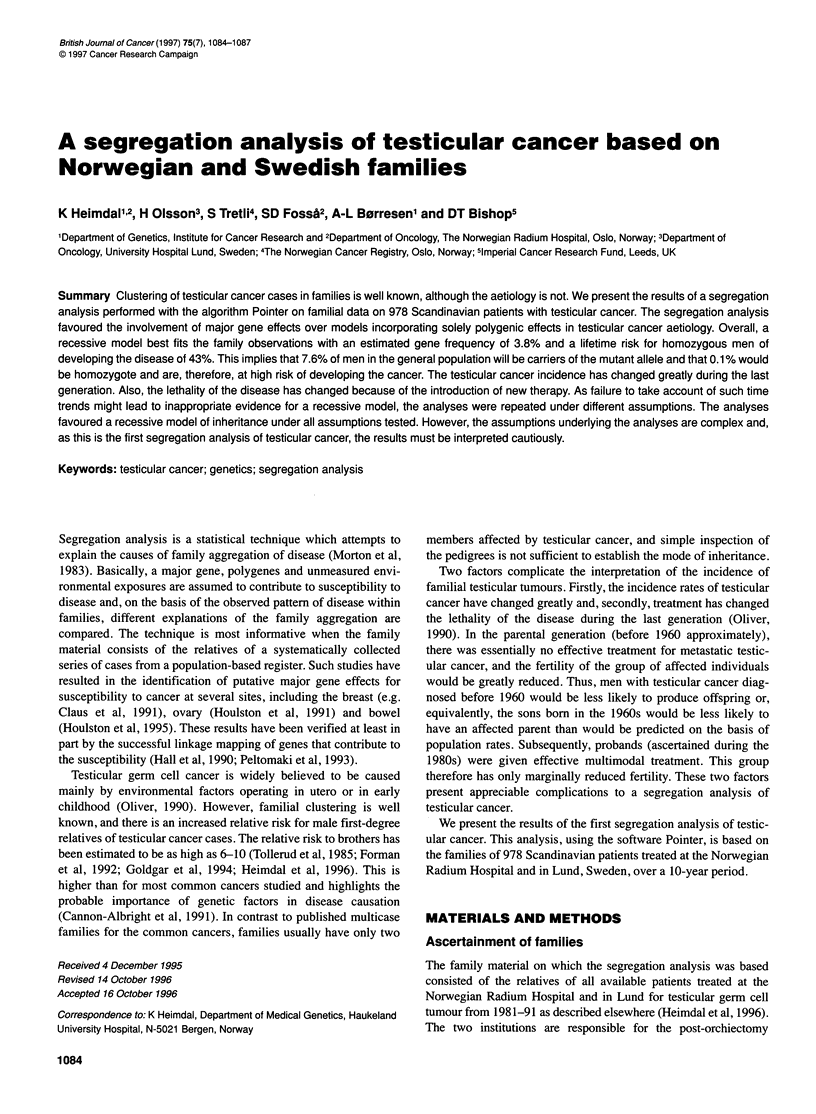

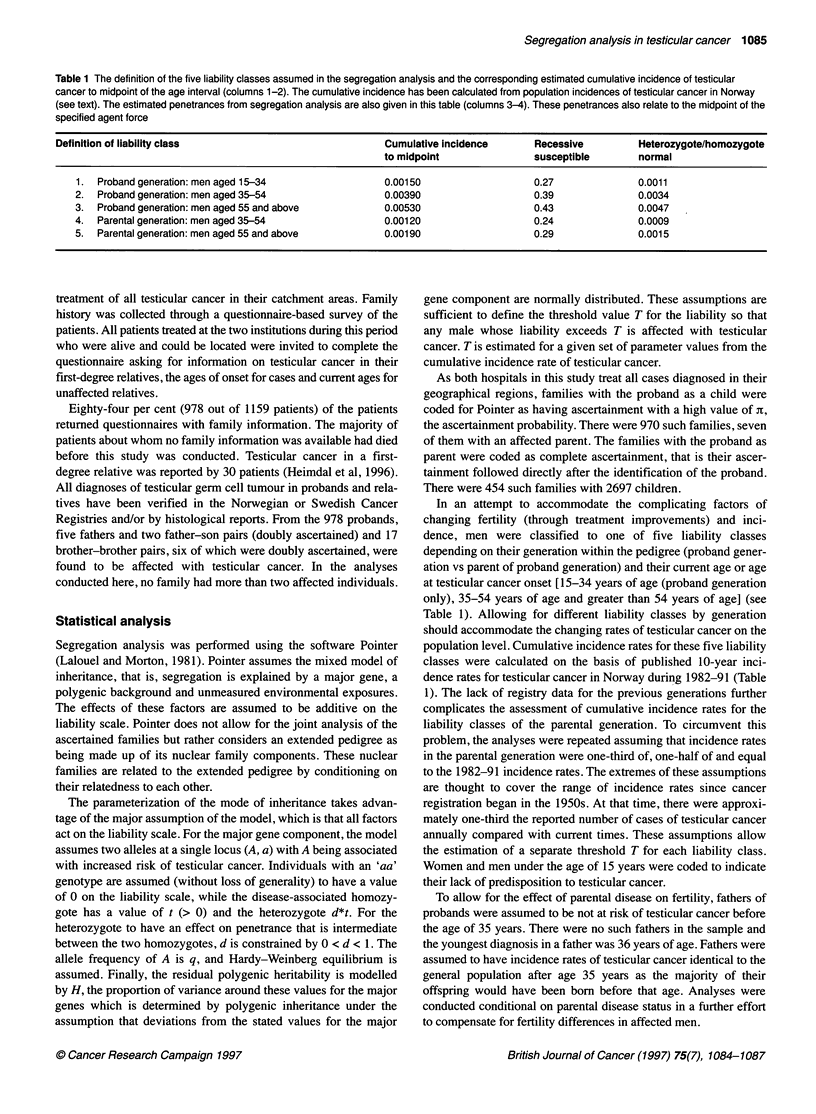

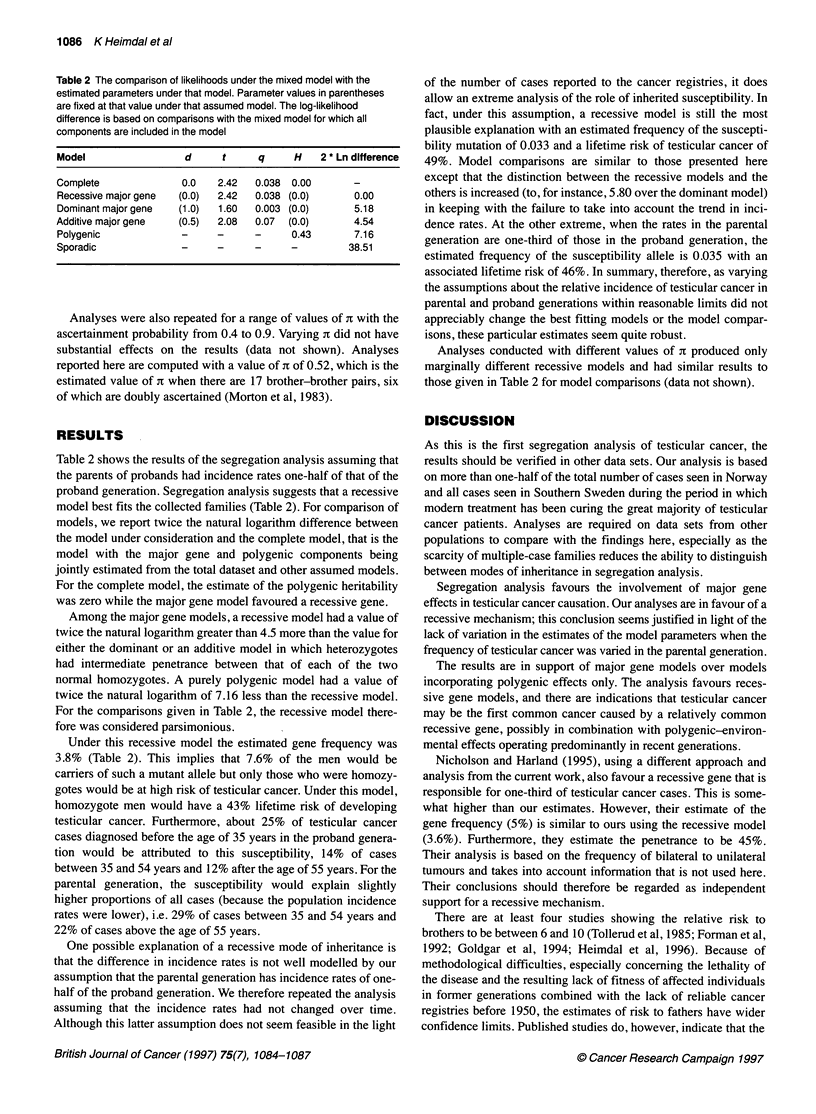

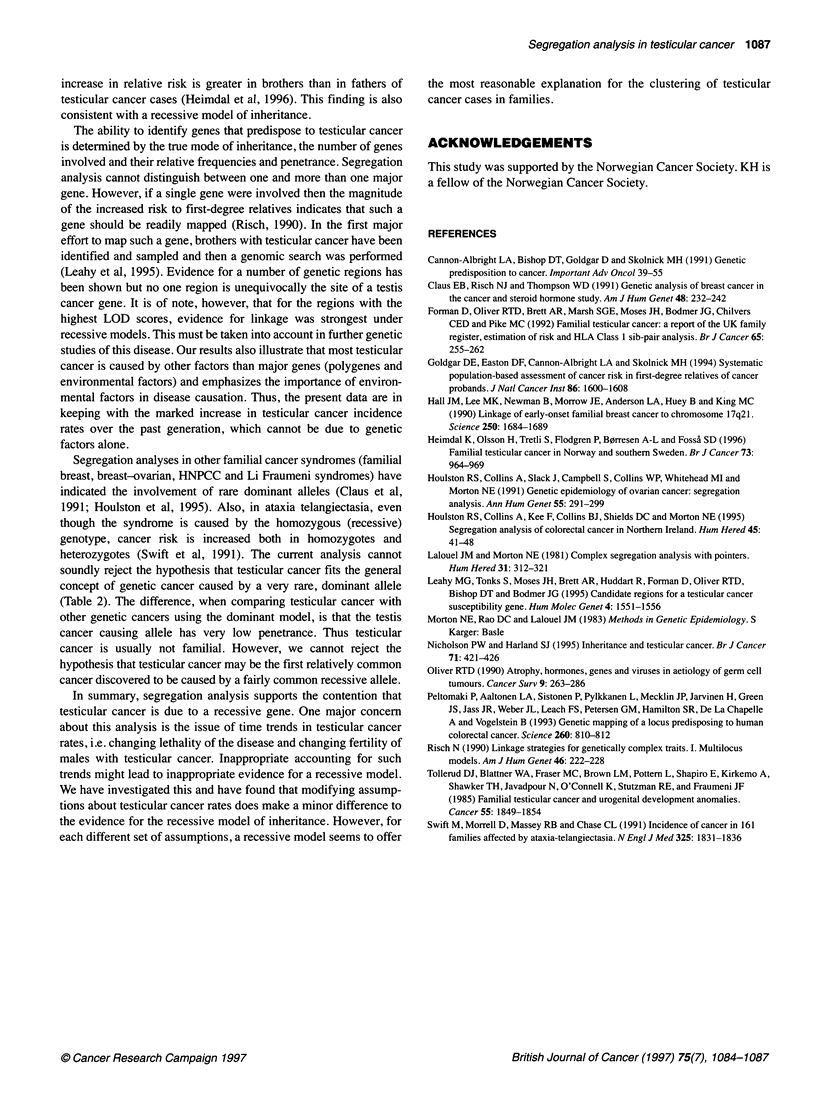


## References

[OCR_00425] Claus E. B., Risch N., Thompson W. D. (1991). Genetic analysis of breast cancer in the cancer and steroid hormone study.. Am J Hum Genet.

[OCR_00433] Forman D., Oliver R. T., Brett A. R., Marsh S. G., Moses J. H., Bodmer J. G., Chilvers C. E., Pike M. C. (1992). Familial testicular cancer: a report of the UK family register, estimation of risk and an HLA class 1 sib-pair analysis.. Br J Cancer.

[OCR_00439] Goldgar D. E., Easton D. F., Cannon-Albright L. A., Skolnick M. H. (1994). Systematic population-based assessment of cancer risk in first-degree relatives of cancer probands.. J Natl Cancer Inst.

[OCR_00444] Hall J. M., Lee M. K., Newman B., Morrow J. E., Anderson L. A., Huey B., King M. C. (1990). Linkage of early-onset familial breast cancer to chromosome 17q21.. Science.

[OCR_00449] Heimdal K., Olsson H., Tretli S., Flodgren P., Børresen A. L., Fossa S. D. (1996). Familial testicular cancer in Norway and southern Sweden.. Br J Cancer.

[OCR_00459] Houlston R. S., Collins A., Kee F., Collins B. J., Shields D. C., Morton N. E. (1995). Segregation analysis of colorectal cancer in Northern Ireland.. Hum Hered.

[OCR_00454] Houlston R. S., Collins A., Slack J., Campbell S., Collins W. P., Whitehead M. I., Morton N. E. (1991). Genetic epidemiology of ovarian cancer: segregation analysis.. Ann Hum Genet.

[OCR_00464] Lalouel J. M., Morton N. E. (1981). Complex segregation analysis with pointers.. Hum Hered.

[OCR_00468] Leahy M. G., Tonks S., Moses J. H., Brett A. R., Huddart R., Forman D., Oliver R. T., Bishop D. T., Bodmer J. G. (1995). Candidate regions for a testicular cancer susceptibility gene.. Hum Mol Genet.

[OCR_00481] Oliver R. T. (1990). Atrophy, hormones, genes and viruses in aetiology germ cell tumours.. Cancer Surv.

[OCR_00485] Peltomäki P., Aaltonen L. A., Sistonen P., Pylkkänen L., Mecklin J. P., Järvinen H., Green J. S., Jass J. R., Weber J. L., Leach F. S. (1993). Genetic mapping of a locus predisposing to human colorectal cancer.. Science.

[OCR_00491] Risch N. (1990). Linkage strategies for genetically complex traits. I. Multilocus models.. Am J Hum Genet.

[OCR_00501] Swift M., Morrell D., Massey R. B., Chase C. L. (1991). Incidence of cancer in 161 families affected by ataxia-telangiectasia.. N Engl J Med.

[OCR_00495] Tollerud D. J., Blattner W. A., Fraser M. C., Brown L. M., Pottern L., Shapiro E., Kirkemo A., Shawker T. H., Javadpour N., O'Connell K. (1985). Familial testicular cancer and urogenital developmental anomalies.. Cancer.

